# Patients' Perceptions and Knowledge of Diabetes and Medications: Implications for Medication Adherence and Glycemic Control in Type 2 Diabetes Patients, Northern Ethiopia

**DOI:** 10.1155/2024/3652855

**Published:** 2024-11-08

**Authors:** Fikadu Hadush, Gebremedhin Beedemariam, Mesfin Haile Kahissay, Shivani A. Patel, Bruck Messele Habte

**Affiliations:** ^1^School of Pharmacy, College of Health Sciences, Addis Ababa University, Addis Ababa, Ethiopia; ^2^Institute of Health Policy Management and Evaluation, University of Toronto, Toronto, Canada; ^3^Rollins School of Public Health, Emory University, Atlanta, Georgia, USA

**Keywords:** diabetes knowledge, glycemic control, medication adherence, medication beliefs, Type 2 diabetes mellitus (T2DM)

## Abstract

Globally, adherence to Type 2 diabetes mellitus (T2DM) medications remains suboptimal. There are limited insights, however, on this issue in the northern region of Ethiopia. This cross-sectional study at Alamata General Hospital investigated the interplay between patients' medication beliefs, diabetes knowledge, adherence, and glycemic control. Data collection was done using structured questionnaires and chart reviews, while descriptive and inferential statistics were for the analysis. Among 305 T2DM patients, poor medication adherence was prevalent (44.6%), alongside suboptimal glycemic control (75.7%). Patients diagnosed for over a decade had an adjusted odds ratio (AOR) of 3.87 for nonadherence, while high concern about medication side effects was associated with a 20.63-fold higher nonadherence risk (AOR = 20.63). Low disease awareness increased nonadherence risk by 4.54 times (AOR = 4.54), while a strong belief in medication necessity was protective (AOR = 0.21). Poor glycemic control was associated with educational background, diabetes awareness, monthly income, and treatment modality. Urgently needed are tailored diabetes education programs in Northern Ethiopia to counteract high rates of poor medication adherence (AOR = 3.87) and glycemic control among T2DM patients. Targeted interventions, emphasizing knowledge enhancement and reinforcing positive beliefs, are essential for improving outcomes in this population.

## 1. Introduction

Diabetes, a chronic metabolic disorder, has emerged as a critical global public health concern, consistently ranking among the Top 4 noncommunicable diseases worldwide. The prevalence of diabetes, notably Type 2 diabetes mellitus (T2DM), has been on a relentless rise, with particularly rapid increases in the past two decades in low- and middle-income countries [1]. Uncontrolled diabetes, or hyperglycemia, plays a central role in driving both macrovascular and microvascular complications [2]. Yet, despite the availability of effective glucose-lowering medication, approximately 45% of T2DM patients exhibit poor glycemic control, with medication nonadherence playing a substantial contributory role [3].

Despite numerous research endeavors, effective management of diabetes remains a significant challenge, with medication adherence standing out as a pervasive issue, especially for patients grappling with chronic diseases like diabetes [4].

In Ethiopia, diabetes prevalence ranges from 2.0% to 6.5%, according to reviewed studies [5]. Beliefs about medicines have emerged as pivotal determinants of medication adherence, with specific necessity and specific concern beliefs exhibiting significant associations [6]. Additionally, there is a noticeable gap in diabetes disease knowledge among the Ethiopian population [7]. Effective diabetes self-management, including education, is recognized as a cornerstone for improving outcomes in individuals with diabetes [8].

In Ethiopia, where the prevalence of diabetes varies between urban and rural areas, several studies have identified a high prevalence of complications linked to suboptimal glycemic control [9, 10]. However, despite these studies shedding light on factors influencing glycemic control including low medication adherence, there remains a paucity of data, particularly in the northern region of Ethiopia, regarding the status of T2DM patients' glycemic control, medication adherence, and the influence of diabetes knowledge and related patient beliefs. This study thus had the objective of assessing patients' diabetes-related knowledge and beliefs on medication adherence and glycemic control among T2DM patients at Alamata General Hospital (AGH) in Northern Ethiopia.

## 2. Materials and Methods

### 2.1. Study Setting, Design, and Period

This study was part of the graduate work of the first author and used a hospital-based cross-sectional study design [11] that was conducted from September to December 2019 among T2DM patients who were on follow-up at AGH, which is one of the public hospitals in Alamata town, in the northern part of the country. This 120-bed hospital is staffed with 246 health personnel and provides in- and outpatient and emergency services to a one million catchment population. It runs a once-a-week chronic care clinic serving diabetes patients using an internist, a general practitioner, and a nurse.

### 2.2. Population

All T2DM patients on follow-up and visiting the chronic care service of AGH were the source population. The study population was all T2DM patients on follow-up and visiting the chronic care service of AGH during the study period. The criteria to include study participants were patients' age greater than or equal to 18, T2DM diagnosis, complete medical records, three consecutive fasting blood glucose (FBG) measurements from the last visit, and willingness to participate.

### 2.3. Sampling Technique and Sample Size Determination

Sample size determination employed Cochran's sample size formula with assumptions of a 45.2% proportion of medication nonadherence [12], a 5% confidence interval width, and a 95% confidence level [13]. A correction formula was applied considering the 1200 T2DM patients treated in the hospital and a 10% nonresponse rate to get 317 T2DM patients, who were consecutively recruited during their follow-up visits. The systematic random sampling technique was employed to select the potential participants until the required sample size was obtained.

### 2.4. Study Variables and Measurements

The dependent variables were medication adherence and glycemic control. The independent variables were sociodemographic variables (age, sex, marital status, educational status, income, religion, marital status, and occupational status), patient and clinical characteristics (smoking, alcohol consumption, chewing khat^1^, duration of diabetes, medication type, and comorbidity presence), diabetes disease knowledge, and belief about medicines (general and antidiabetic medicines specific belief about medicines).

#### 2.4.1. Medication Adherence Rating Scale (MARS)

The MARS is a tool developed in 1999 with 10 yes/no questions. It assesses medication adherence by covering aspects like behavior, attitude toward medication, side effects, and attitudes toward antidiabetic medications. It helps identify barriers to medication adherence. Adherent individuals answer “yes” to Questions 1–6, 9, and 10 and “no” to Questions 7 and 8, with scores ranging from 0 to 10 [14]. It has strong psychometric properties, validated in a large-scale study [15]. MARS is commonly used to measure medication adherence in diabetes patients [16], with a reliability of 0.79 based on Cronbach's alpha [17]. A score of ≥ 80% indicates adherence, while < 80% suggests nonadherence [18].

#### 2.4.2. Beliefs About Medicines

The Beliefs about Medication Questionnaire (BMQ) is a reliable tool for assessing patients' beliefs regarding medications. It comprises two sections: specific beliefs about antidiabetic medications, divided into specific necessity and specific concern, each with five statements rated on a 5-point Likert scale (score range 5–25), and general beliefs about medications, with General Overuse and Harm subscales, each containing four statements rated on the same Likert scale (score range 4–20). High scores on General Overuse indicate perceived overprescribing, while high scores on Harm reflect negative views of medications in general [19]. In this study, high necessity belief was defined as scores ≥ 20 regarding the relevance of antidiabetic medications for their current and future health using this instrument. Respondents who expressed concern about the adverse consequences of their medication (scores ≥ 20) were classified as having a high concern belief.

#### 2.4.3. Michigan Brief Diabetes Knowledge Test

The Diabetes Knowledge Scale which had the objective of assessing patients' knowledge about diabetes care was published by the Michigan Diabetes Research Center in 2016 G.C [20]. The Michigan Simplified Diabetes Knowledge Scale (MSDKS) which contains 20 statements requiring true or false responses was deemed appropriate for the present study. The MSDKS which could be employed for more diverse populations including those with low literacy has been reliably used to assess different aspects of patients' diabetes knowledge including those related to self-care activities [21]. For this study, Good Diabetes Disease Knowledge was defined as a score of ≥ 65% on the MSDKS [21].

Poor glycemic control was defined as an average FBG < 70 mg/dL or > 130 mg/dL, while good glycemic control was defined as an average FBG measurement between 70 and 130 mg/dL [22].

#### 2.4.4. Demographic and Clinical Information

Demographic and clinical data were collected from patients through face-to-face interviews using a structured questionnaire pertaining to age, sex, educational background, and personal behaviors, among others. Additionally, certain clinical details were extracted from medical charts using a structured data abstraction format. These clinical details encompassed the duration of diabetes since diagnosis, the current antidiabetes medications being taken, the presence of any comorbidities, and three consecutive FBG readings.

### 2.5. Data Collection Tools and Procedures

Data was collected from selected participants and their respective medical charts by trained BSc nurses using the abovementioned tools. Quality during the data collection was assured using different techniques, including training of the data collectors, pretesting the study instruments and data collection procedures, and close supervision and feedback by the first author.

### 2.6. Data Analysis

Data analysis was carried out using SPSS software Version 25. Descriptive statistics was first used to present the categorical data in the form of frequencies and percentages for categorical data, while mean and standard deviation were employed for normally distributed continuous data. Inferential statistics was used to assess factors that are associated with the dependent variables, medication adherence, and glycemic control. Independent variables with *p* values ≤ 0.25 in the binary model were included in the multivariable logistic regression model. Those variables demonstrating *p* ≤ 0.05 and 95% CI were considered to be significantly associated with the dependent variables. This study followed STROBE guidelines to report the findings [23].

### 2.7. Ethical Considerations

The study protocol was ethically approved by the School of Pharmacy, College of Health Sciences, Addis Ababa University (AAU) Ethics Review Committee (ERB/SOP/59/04/2019). In addition, participant informed consent, confidentiality, and anonymity were maintained during data collection and reporting.

## 3. Results

### 3.1. Demographic and Clinical Characteristics of Patients

Among the total of 317 patients that were included in the study, 305 patients completed the study. Among these participants, half were male (51.2%) and had no formal education (53.1%), two-thirds were married (66%), while nearly three-quarters and more than half (57.1%), respectively, were followers of the Orthodox Christian faith and had a household monthly income of less than or equal to $75 (equivalent to 2275 Ethiopian Birr), as depicted in Table 1. The mean age of participants was 56.5 ± 12.4 years with maximum and minimum ages ranging from 30 to 85 years. Surprisingly, none reported having received any diabetes-related health education in the previous year.

The clinical profile of the study participants reveals several noteworthy trends. Approximately one in 10 (12.5%) and one in 20 (5.6%) of the study participants were daily alcohol consumers and khat users, respectively. Less than one-third (30.5%) of the study participants had comorbid conditions, with hypertension (24.3%) being the most common, followed by HIV (5.2%). Among the participants, 71.8% were exclusively taking oral hypoglycemic agents, with metformin (34.1%) being the most frequently prescribed antidiabetic medication (see Table 2).

### 3.2. Medication Adherence Pattern and Predictors of Poor Adherence

Among the study participants, 44.6% were nonadherent to their recommended antidiabetic regimens, with the mean adherence score being 7.7 ± 1.6 (see Table 3). Study participants who have been diagnosed with diabetes for more than 10 years compared with those less than 5 years (AOR = 3.87, 95% CI [1.48–10.07]), had high concern about the negative effects of their medications (AOR = 20.63, 95% CI [5.15–82.61]), and had low awareness about diabetes (AOR = 4.54, 95% CI [1.53–13.46]) were more likely to be nonadherent to their antidiabetic medications, while those with a high belief toward their antidiabetic medication necessity (AOR = 0.21, 95% CI [0.11–0.40]) were less likely to be nonadherent to their medications, as can be seen on Table 4.

### 3.3. Poor Glycemic Control and Contributing Factors

**Figure 1:**
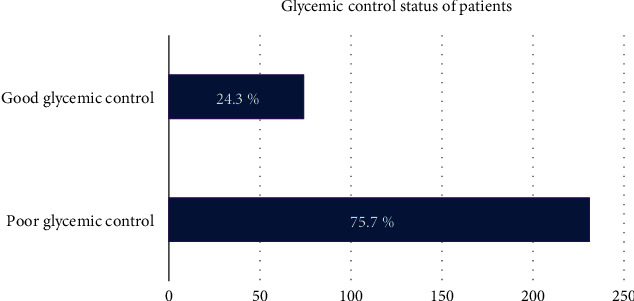
Glycemic control level of T2DM patients on follow-up at Alamata General Hospital, Alamata, Ethiopia, September to December 2019 (*N* = 305).

Among the study participants, three-quarters (75.7%) had poor glycemic control, as shown in Figure 1. Participants who were high school students (AOR = 5.54, 95% CI [1.11–27.60]) and TVET/diploma holders (AOR = 9.27, 95% CI [1.98–43.36]) compared with those who had no formal education and had poor awareness toward their diabetes (AOR = 4.34, 95% CI [1.74–10.81]) were more likely to have poor glycemic control while those had a monthly household income of greater than or equal to $150.05 (AOR = 0.18, 95% CI [0.06–0.48]) compared to those with monthly income of $75 and those that are a combined oral antidiabetic agents and insulin injections (AOR = 0.33, 95% CI [0.15–0.71]) were less likely to have poor glycemic control compared to those on oral antidiabetic agents only (see Table 5).

## 4. Discussion

The findings of this study revealed suboptimal adherence to antidiabetic medications and glycemic control were common among the study participants. Significant predictors of medication nonadherence were duration of diabetes, beliefs about medications, and awareness about their diabetes. On the other hand, participants' education level, economic status, type of medication regimen, and diabetes-related knowledge were significant predictors of glycemic control.

Close to half of the study participants, that is, 44.6%, reported nonadherence to their antidiabetic medication regimen which was similar to those reported from other studies reported from Ethiopia [12] and elsewhere [24] but higher nonadherence compared to those reported in Southwest Ethiopia (24.9%) [25] and Addis Ababa (33.2%) [26]. The variation in adherence rates may be attributed to differences in assessing medication adherence, the educational backgrounds of participants, and methodological variances. Importantly, the high levels of suboptimal adherence are concerning, which in turn could lead to poor glycemic control, associated morbidity and mortality, and increased healthcare costs [2, 27].

Low awareness about diabetes has been identified as a significant determinant of both suboptimal adherence to medications and glycemic control similar to those reported by other studies [7, 28–30]. This finding is therefore indicative of the need to organize diabetes education for patients in a manner that addresses individual and social contexts. The provision of education for example should consider patients' educational levels to provide appropriate diabetes education. It is also important that providers should not assume that patients with longer duration no longer need education and instead should regularly assess needs and provide needs-based education.

Another significant factor that influences diabetes outcomes is the low household monthly income, similar to reports by other studies [31]. This finding is relevant in Ethiopia, where a large majority of the population has low income. This requires routine assessment of medicine access issues and, when appropriate, the need to provide support so that patients can access a community-based health insurance scheme to enhance healthcare access and glycemic control [32]. This, of course, requires policy-level interventions to expand and strengthen community and other health insurance schemes to reach the needs of low-income groups of the population.

With regard to diabetes education, adequate attention should be given to the antidiabetic medications that have been reported to significantly affect adherence. Accordingly, high necessity beliefs were positively associated with better adherence, while high concerns about medications have been associated with poor adherence, as have been reported by other studies in different parts of the world [24, 33] and Ethiopia [34]. This is further evidence to give due emphasis to the necessity of antidiabetic medications alongside other nonpharmacological treatment regimens on the one hand and alleviate unfounded concerns about the short- and long-term concerns about these medications. In addition, providers should make their patients aware early on of the fact that diabetes is a lifelong condition that may progress over time and thus requires medications on a chronic basis, including insulin, at some point in time [35].

## 5. Limitations

This study had limitations in that a cross-sectional study design was used, which may limit the ability to establish causal inferences between independent and dependent variables. In addition, the self-reported measure used to assess medication adherence may overestimate adherence. It should be noted, however, that this has previously been validated to assess adherence. In any case, the findings should be interpreted in light of these limitations.

## 6. Conclusions and Recommendations

This study revealed that unacceptably high levels of the participants reported poor adherence to antidiabetic medications which were associated with low awareness about diabetes, high concerns about medications, and a long duration of diabetes diagnosis. Furthermore, a large proportion of the participants had poor glycemic control, which in turn was associated with low awareness about diabetes, low economic status, and relatively high educational status. These findings are indicative of the need to provide needs-based education to the patients regarding diabetes and the medications on the one hand but also consider low medication access issues and provide support in that regard when needed. The latter intervention may evidently require policy-level interventions in terms of expanding and strengthening community-based and other insurance schemes to cater to the low-income sects of the population.

## Figures and Tables

**Table 1:** Sociodemographic characteristics of T2DM patients on follow-up at Alamata General Hospital, Alamata, Ethiopia, September to December 2019 (*N* = 305).

**Variable**	**n** ** (%)**
Sex	
Male	156 (51.15)
Female	149 (48.85)
Age (in years)	
30–39	29 (9.51)
40–49	65 (21.31)
50–59	84 (27.54)
60–69	66 (21.64)
≥ 70	61 (20)
Religion	
Orthodox	225 (73.77)
Muslim	78 (25.57)
Catholic and Protestant	2 (0.66)
Marital status	
Single	6 (1.97)
Married	203 (66.56)
Divorced	47 (15.41)
Widowed	49 (16.06)
Educational status	
No formal education	162 (53.11)
Primary school	61 (20)
High school	24 (7.87)
TVET/diploma	35 (11.48)
Degree and above	23 (7.54)
Occupation	
Government	57 (18.69)
Merchant	70 (22.95)
Farmer	94 (30.82)
Housewife	19 (6.23)
Private	18 (5.90)
Unemployed	9 (2.95)
Daily labor	6 (1.97)
Others	32 (10.49)
Household monthly income (USD)	
≤ 75.00	174 (57.05)
75.05–150	50 (16.39)
≥ 150.05	81 (26.56)

*Note:* Others = retired, house rent, student, maid, and sharia court, absolute poverty line = 598.67 birr per month, real per adult total consumption expenditure average birr = 1032.58 per month, adult equivalent household size = 3.8.

Abbreviation: TVET = technic and vocational education and training.

**Table 2:** Clinical characteristics of T2DM patients on follow-up at Alamata General Hospital, Alamata, Ethiopia, September to December 2019 (*N* = 305).

**Variable**	**N** ** (%)**
Smoking status	
Not at all	287 (94.10)
Occasionally	14 (4.59)
Daily	4 (1.31)
Alcohol use	
Daily	38 (12.46)
Not at all	194 (63.61)
Occasionally	73 (23.93)
Khat use	
Daily	17 (5.57)
Not at all	264 (86.56)
Occasionally	24 (7.87)
Duration of diabetes (years)	
< 5	173 (56.72)
5–10	87 (28.53)
> 10	45 (14.75)
Comorbidities	
No comorbidity	212 (69.51)
Hypertension	74 (24.26)
HIV	16 (5.25)
Others	3 (0.98)
Type of antidiabetes medication	
OHA	219 (71.80)
OHA and insulin injection	53 (17.38)
Insulin injection	33 (10.82)

*Note:* Others = asthma, tuberculosis, and hyperlipidemia; occasionally = during special conditions or irregularly; khat = khat is a stimulant plant native to Ethiopia, known for its leaves and shoots that induce euphoria and alertness when consumed, but it is associated with health and social issues.

Abbreviations: HIV = human immunodeficiency virus, OHA = oral hypoglycemic agent.

**Table 3:** Distribution of items in the Medication Adherence Rating Scale (MARS) of T2DM patients on follow-up at Alamata General Hospital, Alamata, Ethiopia, September to December 2019 (*N* = 305).

**S. No.**	**Frequencies of responses on the MARS item**	**Adherent, ** **n** ** (%)**
1	Do you ever forget to take your antidiabetic medication?	189 (61.97%)
2	Are you careless at times at taking your antidiabetic medication?	274 (89.84%)
3	When you feel better, do you sometimes stop taking your antidiabetic medication?	276 (90.49%)
4	Sometimes if you feel worse when you take the antidiabetic medication, do you stop taking it?	281 (92.13%)
5	I take my antidiabetic medication only when I am sick.	301 (98.69%)
6	It is unnatural for my mind and body to be controlled by antidiabetic medication.	141 (46.23)
7	My thoughts are clearer on antidiabetic medication.	241 (79.02%)
8	By staying on antidiabetic medication, I can prevent getting sick.	273 (89.51%)
9	I feel weird, like a zombie, on antidiabetic medication.	177 (58.03%)
10	Antidiabetic medication makes me feel tired and sluggish.	188 (61.64%)

*Note:* Adherent = “no” response for Q 1–6, 9, and 10 and “yes” response for Q 7 and 8; range =1–10.

**Table 4:** Multivariable logistic regression analysis of predictors of poor medication adherence in T2DM patients at Alamata General Hospital, Alamata, Ethiopia, September to December 2019 (*N* = 305).

**Variable**	**Status of medication adherence, ** **n** ** (%)**				
	**Adequate medication adherence (** **M** **A** **R** **S** > 80** %)**	**Poor medication adherence (** **M** **A** **R** **S** ≤ 80** %)**	**COR for poor medication adherence (95% CI)**	**AOR for poor medication adherence (95% CI)**	**p** ** value**
Duration of diabetes					
< 5 years	106 (61.27)	67 (38.73)	Reference	Reference	
5–10 years	43 (49.43)	44 (50.57)	1.62 (0.96–2.72)	1.87 (0.92–3.78)	0.084
> 10 years	20 (44.44)	25 (55.56)	1.98 (1.02–3.84)	3.87 (1.48–10.07)	0.006⁣^∗^
Specific necessity					
Low necessity	29 (30.21)	67 (69.79)	Reference	Reference	
High necessity	140 (66.99)	69 (33.01)	0.21 (0.13–0.36)	0.21 (0.11–0.40)	< 0.001⁣^∗^
Specific concern					
Low concern	165 (58.72)	116 (41.28)	Reference	Reference	
High concern	4 (16.67)	20 (83.33)	7.11 (2.37–21.36)	20.63 (5.15–82.61)	< 0.001⁣^∗^
Diabetes disease knowledge					
Good knowledge	40 (83.33)	8 (16.67)	Reference	Reference	
Poor knowledge	129 (50.19)	128 (49.81)	4.96 (2.24–11.01)	4.54 (1.53–13.46)	0.006⁣^∗^

Abbreviations: AOR = adjusted odds ratio, COR = crude odds ratio.

⁣^∗^Significant factors.

**Table 5:** Factors independently associated with poor glycemic control in T2DM patients at Alamata General Hospital, Alamata, Ethiopia, September to December 2019 (*N* = 305).

**Variables**	**Glycemic level**		**COR for poor control (95% CI)**	**AOR for poor control (95% CI)**	**p** ** value**
	**Good control (FBG 70–130 mg/dL), ** **n** ** (%)**	**Poor control (** **F** **B** **G** < 70 **mg/dL or FBG** > 130 **mg/dL), n (%)**			
Education					
No formal education	31 (19.14)	131 (80.86)	Reference	Reference	
Primary school	20 (32.79)	41 (67.21)	0.49 (0.25–0.94)	1.03 (0.43–2.47)	0.955
High school	3 (12.50)	21 (87.50)	1.66 (0.46–5.91)	5.54 (1.11–27.60)	0.037⁣^∗^
TVET/diploma	8 (22.86)	27 (77.1 4)	0.8 (0.33–1.93)	9.27 (1.98–43.36)	0.005⁣^∗^
Degree and above	12 (52.17)	11 (47.83)	0.22 (0.09–0.54)	2.53 (0.52–12.40)	0.252
Household monthly income (ETB)					
≤ 75.00	33 (18.97)	141 (81.03)	Reference	Reference	
75.05–150	6 (12)	44 (88)	1.72 (0.68–4.36)	1.26 (0.42–3.80)	0.686
≥ 150.05	35 (43.21)	46 (56.79)	0.31 (0.17–0.55)	0.18 (0.06–0.48)	0.001⁣^∗^
Type of antidiabetes medication					
OHA	47 (21.46)	172 (78.54)	Reference	Reference	
OHA and insulin	19 (35.85)	34 (64.15)	0.49 (0.26–0.93)	0.33 (0.15–0.71)	0.005⁣^∗^
Insulin only	8 (24.24)	25 (75.76)	0.85 (0.36–2.02)	0.52 (0.17−1.55)	0.241
Diabetes disease knowledge					
Good knowledge	23 (47.92)	25 (52.08)	Reference	Reference	
Poor knowledge	51 (19.84)	206 (80.16)	3.72 (1.95–7.08)	4.34 (1.74–10.81)	0.002⁣^∗^

⁣^∗^Significant factors contributing to poor glycemic control.

## Data Availability

The datasets generated during and/or analyzed during the current study are available from the corresponding author on reasonable request.

## Endnotes

^1^An evergreen plant with the scientific name *Catha edulis* that contains psychoactive substances.
